# Purinergic implication in amyotrophic lateral sclerosis—from pathological mechanisms to therapeutic perspectives

**DOI:** 10.1007/s11302-018-9633-4

**Published:** 2018-11-14

**Authors:** M. Cieślak, K. Roszek, M. Wujak

**Affiliations:** 1 Neurology Clinic, Marek Cieślak, Toruń, Poland; 2 Department of Biochemistry, Faculty of Biology and Environmental Protection, Nicolaus Copernicus University in Toruń, 1 Lwowska St, 87-100 Toruń, Poland

**Keywords:** ALS, Purinergic signaling, ATP, Neuroinflammation, Motor neuron degeneration, Neuroglial activation

## Abstract

Amyotrophic lateral sclerosis (ALS) is a clinically heterogeneous disorder characterized by degeneration of upper motor neurons in the brainstem and lower motor neurons in the spinal cord. Multiple mechanisms of motor neuron injury have been implicated, including more than 20 different genetic factors. The pathogenesis of ALS consists of two stages: an early neuroprotective stage and a later neurotoxic. During early phases of disease progression, the immune system through glial and T cell activities provides anti-inflammatory factors that sustain motor neuron viability. As the disease progresses and motor neuron injury accelerates, a rapidly succeeding neurotoxic phase develops. A well-orchestrated purine-mediated dialog among motor neurons, surrounding glia and immune cells control the beneficial and detrimental activities occurring in the nervous system. In general, low adenosine triphosphate (ATP) concentrations protect cells against excitotoxic stimuli through purinergic P2X4 receptor, whereas high concentrations of ATP trigger toxic P2X7 receptor activation. Finally, adenosine is also involved in ALS progression since A2A receptor antagonists prevent motor neuron death. Given the complex cellular cross-talk occurring in ALS and the recognized function of extracellular nucleotides and adenosine in neuroglia communication, the comprehensive understanding of purinome dynamics might provide new research perspectives to decipher ALS and help to design more efficient and targeted drugs. This review will focus on the purinergic players involved in ALS etiology and disease progression and current therapeutic strategies to enhance neuroprotection and suppress neurotoxicity.

## Introduction

Amyotrophic lateral sclerosis (ALS), also known as Charcot’s or Lou Gehring’s disease, is the most common adult-onset motor neuron disorder (MND) characterized by degeneration of motor neurons, leading to progressive paralysis and death [1]. Due to a staggeringly complex etiology, the identification of a principal mechanism and development of an effective therapeutic treatment for ALS still remains a research challenge. In the light of current findings, it has become evident that pathogenesis of ALS is not restricted to motor neurons but attributed to the abnormal interactions of neurons and non-motoneuronal cells such as microglia, astrocytes, interneurons, Schwann and skeletal muscle cells, oligodendrocytes, and possibly endothelial cells and T lymphocytes [2–5]. Therefore, further research efforts to decipher the pathogenesis of ALS should not only focus on potential disease triggers but also on mechanisms for the propagation of pathological processes and signals between individual cells in a non-cell autonomous manner.

Currently, it is postulated that an understanding of the purinergic impact on neuroinflammation underpinning pathology of neurological disorders is essential for the development of efficacious interventions. Extracellular adenosine triphosphate (ATP) and adenosine (Ado) are recognized as the most powerful purinergic signaling molecules, directing intercellular cross-talk and thereby equilibrating beneficial and detrimental activities occurring in the nervous system. In the present work, we will discuss the most relevant research advances in deciphering a role of the purinome in mediating pathological mechanisms underlying dysfunction of neuroglia and consequent motor neuron damage. By presenting the time-specific involvement of diverse purinergic receptors in the disease progression, we will discuss their relevance for the development of new more powerful diagnostic and therapeutic avenues for amyotrophic lateral sclerosis.

## Basic notions of ALS

ALS is characterized by degeneration of motor neurons in the brainstem (upper motor neurons, UMN) and in the spinal cord (lower motor neurons, LMN). This results in a progressive muscle denervation in upper and lower limbs, torso, and bulbar region leading ultimately to weakness and atrophy of skeletal muscles [1]. ALS is classified as a rare adult-onset disease with 58–60 years as average age of onset*.* The incidence rate of ALS is around 1 to 2.6 cases per 100,000 persons annually, whereas the prevalence is approximately 6 cases per 100,000 per year [6]. The vast majority of ALS patients die from respiratory failure within 3–5 years after onset of symptoms, while only 10% survive beyond 10 years [7]. Approximately 90% of ALS cases occur randomly and are termed sporadic ALS (sALS), while the remaining 10% of cases are classified as familial ALS (fALS) having autosomal dominant pattern of inheritance, although some autosomal recessive pedigrees have been reported [8]. Due to a highly complex pathology and heterogeneous clinical presentations, ALS is considered as a multi-genetic, multi-systemic, and multi-factorial disorder [9]. A composite etiology of ALS is caused by the influence of various genetic, biological, and environmental factors. The disease arises as a consequence of multiple pathophysiological mechanisms and cellular perturbations, including oxidative stress [10], neuroinflammation [11], glutamate excitotoxicity [12], mitochondrial dysfunction [13], RNA metabolism impairment [14, 15], protein misfolding and aggregation, and ER stress [16, 17], dysfunction of the ubiquitin–proteasome system [18], lack of trophic (growth) factors [19], aberrant axonal conduction [20], immune system deficiency [21], and blood-spinal cord barrier impairment [22].

**Fig. 1:**
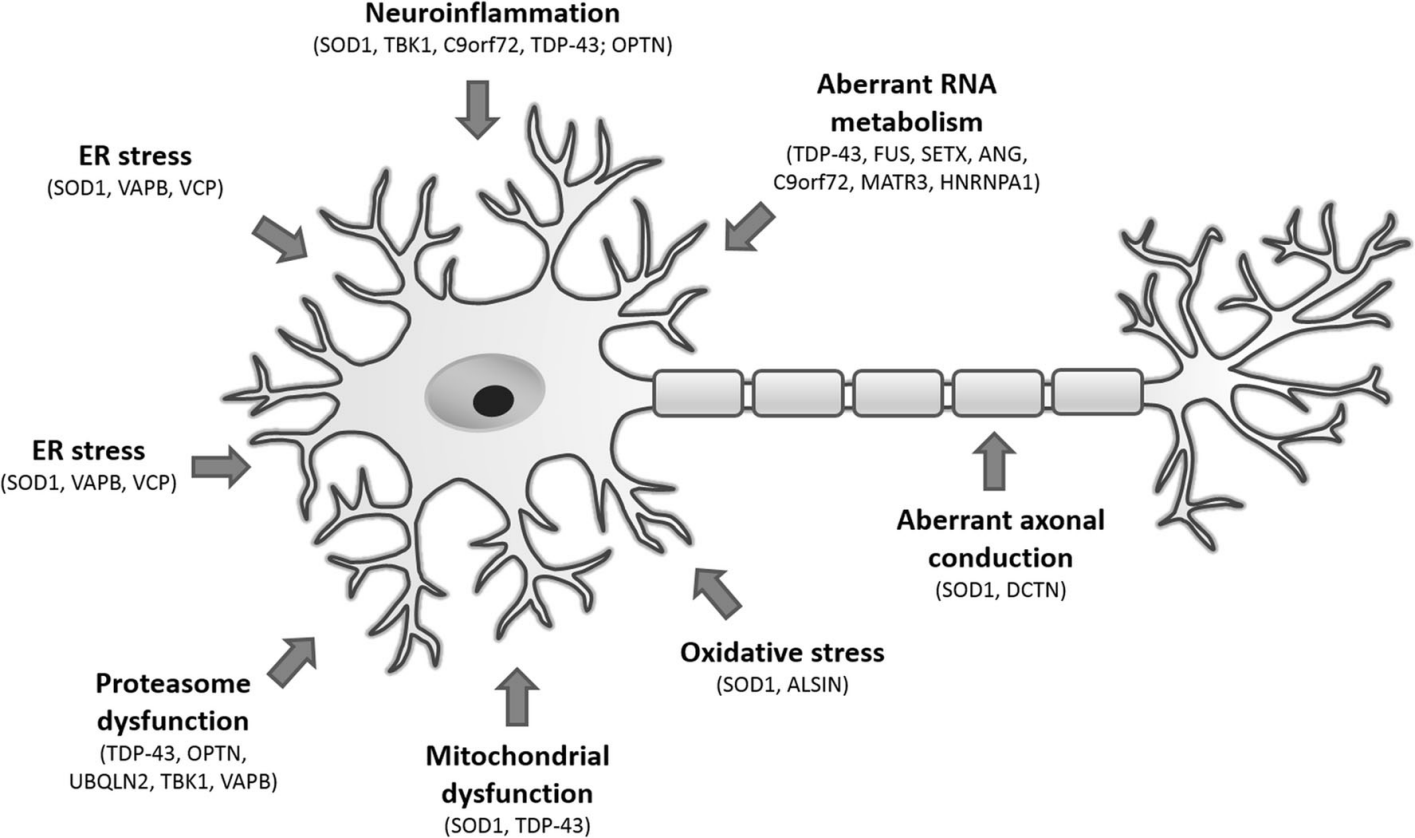
Main key pathogenic mechanisms and cellular perturbations underlying the pathogenesis of ALS, including genetic background

In 1990s, it has been discovered that some cases of fALS are associated with mutations in a gene localized on chromosome 21q21.1 encoding superoxide dismutase 1 (SOD1), also known as Cu/Zn superoxide dismutase [23]. Since then, over 160 mutations distributed throughout the 153-amino acid SOD1 polypeptide have been identified in association with ALS and till date, many cell and animal models expressing exogenous mutant SOD1 have been developed in order to investigate the etiology of ALS [24, 25]. Following the discovery of mutant SOD1, numerous mutations in other genes have been identified to be associated with ALS, thereby opening up new avenues for the disease modeling. Figure 1 presents a brief overview of key cellular mechanisms and genetic mutations contributing to the motor neuron damage in ALS.

## Purinergic players in the central nervous system

Purinergic signaling utilizes nucleotides and nucleosides as signaling molecules to activate two types of membrane-bound receptors. The P1 receptors comprise four subtypes of G protein-coupled receptors (A1, A2A, A2B, A3) that are activated exclusively by adenosine, whereas the P2 receptors are activated by nucleotides (ATP, ADP, UTP, UDP) and due to structural and pharmacological differences are classified into two subtypes: G protein-coupled P2Y receptors (1, 2, 4, 6, 11, 12, 13, 14) and ionotropic P2X receptors (1–7) [26]. Neuroglia and neurons contain different combinations of purinergic receptors, which contributes to a versatile regulation of pathophysiological processes in the NCS (Table 1) [27–43].

**Table 1:** Distribution of P1 and P2 receptors in the nervous system

	Receptor subtype	Distribution/cell type	References
P1 receptors	A1	Widely distributed in brain (ISH); high expression levels in primary cultures of astrocytes from different brain regions, e.g., cerebellum, hippocampus, cortex, thalamus, spinal cord (RB, RT-PCR)	[27, 28]
	A2A	Brain neurons and astrocytes (RB), high expression levels in dopamine-rich regions (IHC), low expression levels in hippocampus (RT-PCR, IHC)	[29, 30]
	A2B	Widely distributed but at low expression levels; glial and neuronal cells (RT-PCR)	[30, 31]
	A3	Neurons of cerebellum and hippocampus (RT-PCR, WB), astrocytes (WB), generally low expression level	[32]
P2X receptors	P2X1	Cerebellum (IHC, RT-PCR), dorsal horn spinal neurons, astrocytes, microglia (IHC)	[33, 34]
	P2X2	Widely distributed in neuronal structures, including the cortex, hippocampus, cerebellum, spinal cord (ISH), autonomic and sensory ganglia neurons (IHC)	[35, 36]
	P2X3	Sensory neurons, nucleus tractus solarius neurons, some sympathetic neurons (ISH, IHC)	[34, 35]
	P2X4	Widely distributed in CNS (ISH), neurons, astrocytes, activated microglia (IHC)	[35, 36]
	P2X5	Neurons in spinal cord, astrocytes (ISH, IHC)	[35]
	P2X6	widely distributed in CNS (ISH); motor neurons in spinal cord (IHC)	[35, 36]
	P2X7	Ependymal cells lining the ventricles (RT-PCR), hippocampus (RT-PCR), microglia, astrocytes, apoptotic cells (IHC, WB)	[37]
P2Y receptors	P2Y1	Widespread distribution in mammalian brain, including the cerebral cortex, hippocampus and cerebellum (IHC), neurons and microglia (IHC, RT-PCR)	[38]
	P2Y2	Astrocytes (IHC, RT-PCR)	[38]
	P2Y4	Brain neurons (IHC, RT-PCR) and microglia (RT-PCR)	[38, 39]
	P2Y6	Activated microglia (ISH, IHC, RT-PCR)	[39–41]
	P2Y11	brain neurons and oligodendrocytes of nucleus accumbens, parahippocampal gyrus, putamen and striatum (RT-PCR)	[39]
	P2Y12	Hippocampal pyramidal neurons (RT-PCR), resting microglia (RT-PCR)	[39]
	P2Y13	Brain (RT-PCR) neurons and oligodendrocytes (IHC), microglia (ISH, IHC)	[40, 42]
	P2Y14	CNS astrocytes (RT-PCR), discrete brain regions (ISH)	[40, 43]

*ISH* in situ hybridization, *RB* radioligand binding, *RT-PCR* reverse transcriptase polymerase chain reaction, *IHC* immunohistochemistry, *WB* Western Blot

**Fig. 2:**
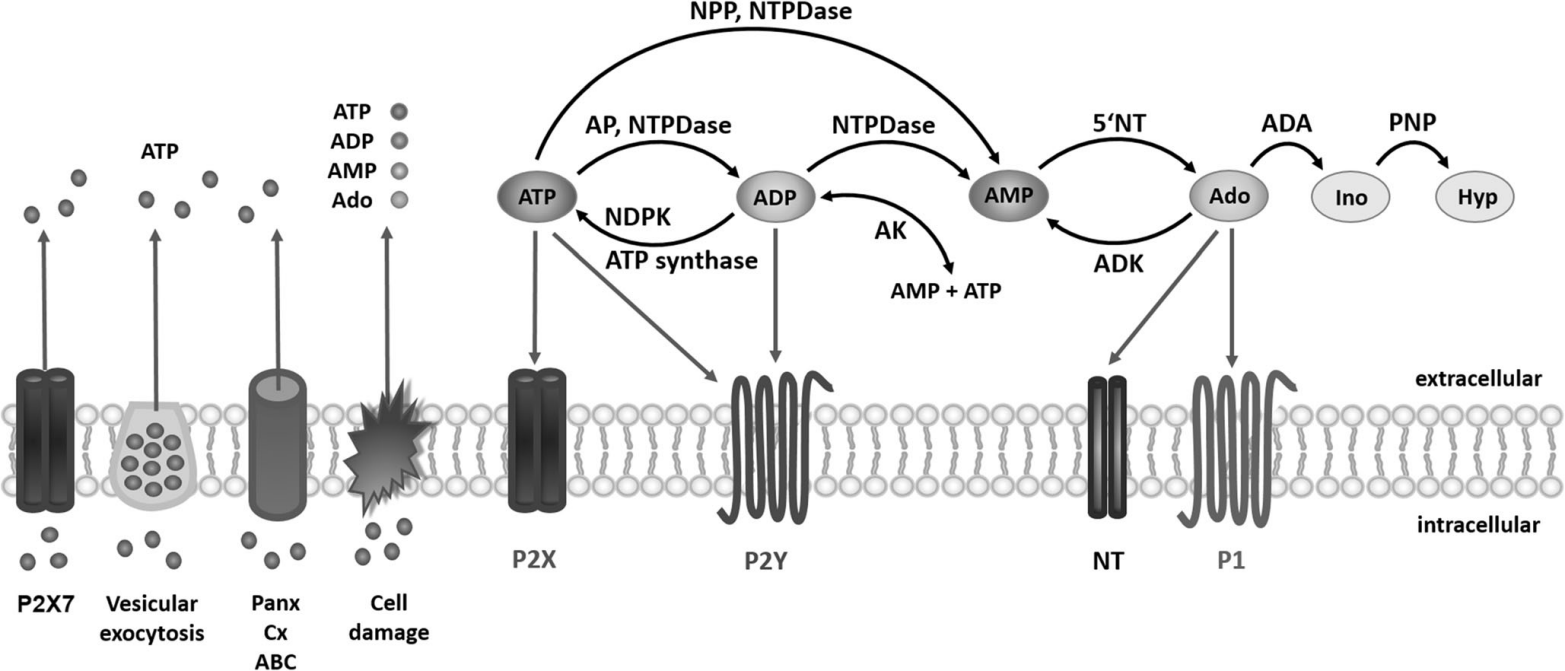
Enzymes involved in the metabolism of extracellular nucleotides and nucleosides. AMP, ADP, and ATP nucleotides breakdown to adenosine (Ado) and other nucleosides such as inosine (Ino) and hypoxanthine (Hyp): nucleotide triphospho-diphosphohydrolases (NTPDases), nucleotide pyrophosphohydrolases/phosphodiesterases (NPP), alkaline phosphatase (AP), 5′-nucleotidase (5′-NT, also known as CD73), adenosine deaminase (ADA), and purine nucleoside phosphorylase (PNP). ATP re-synthesis via backward phosphotransfer reactions: adenylate kinases (AK), nucleoside-diphosphate kinases (NDPK), and ATP synthase. P2X7, pannexin (Panx), connexin (Cx), and ATP-binding cassette (ABC) proteins represent channel and transport-mediated ATP release pathways

The signaling events are triggered upon the release of purines and pyrimidines into the extracellular matrix by exocytosis, facilitated diffusion, through channels, and finally as a result of cell damage and lysis [26]. Extracellular ATP concentration drastically increases in response to diverse biological, chemical, or mechanical stimuli, such as hypoxia, infection, physical trauma, neurodegeneration, and neuroinflammation [44]. To measure ATP in the pericellular space, several in vitro and in vivo methods are available, including a novel pmeLUC system which is a simple and reliable in vivo tool for investigating the dynamic changes of extracellular ATP [45]. After release, the pericellular concentration of ATP rises even up to 100–200 μM and is rapidly reduced to 1–100 nM by the activities of nucleotide-metabolizing enzymes, existing in a membrane-bound or soluble form, with the consequent production of ADP and/or AMP, including nucleotide triphospho-diphosphohydrolases (NTPDases), nucleotide pyrophosphohydrolases/phosphodiesterases (NPP), and alkaline and acid phosphatases (Fig. 2). Finally, AMP hydrolysis catalyzed by 5′-nucleotidase (5′-NT or CD73) results in production of adenosine which is further deaminated via inosine into hypoxanthine by adenosine deaminase (ADA) and purine nucleoside phosphorylase (PNP), respectively [46, 47]. Taken together, the breakdown of ATP by ecto-nucleotidases not solely terminates its extracellular messenger functions but also generates additional agonists, namely ADP and adenosine. It is important to emphasize that the phosphorylation of adenosine to AMP by adenosine kinase (ADK) enters the opposite metabolic pathway whereby adenosine can be converted back to its nucleotide derivatives. The synthesis of ADP and ATP molecules is catalyzed by nucleotide-phosphorylating enzymes such as adenylate kinases (AK) and nucleoside-diphosphate kinases (NDPK) and ATP synthase [47, 48]. Consequently, the interplay between phosphohydrolysis and phosphotransfer reactions provides a precise and dynamic control of the duration, magnitude, and direction of purinergic and pyrimidinergic signals. Most importantly, the existence of many P1/P2 receptor subtypes differing in their sensitivity to agonists and the possibility that the same ligand activates more than one receptor subtype increase the complexity of the purinome and generate an extensive network of overlapping cellular responses [9, 49].

## Cellular players in neuroinflammation

In the central nervous system, ATP acting as a neurotransmitter and neuromodulator regulates a wide array of physiological processes such as neurotransmission, cell-to-cell communication, neurite outgrowth, as well as cell proliferation, migration, differentiation, and apoptosis [26]. ATP is released extracellularly under conditions of tissue stress or injury by microglia, astrocytes, and damaged neurons. The increase in extracellular ATP (eATP) level is recognized by competent cells as damage-associated molecular pattern (DAMP) signal capable of initiating and propagating neuroinflammatory response [3, 50, 51]. The principle functions of neuroinflammation are to limit tissue damage and initiate tissue repair. However, disproportionate neuroinflammatory responses, particularly when chronic, promote apoptosis and necrosis and influence the synaptic and intrinsic membrane properties of neurons [52].

A dominant role for neuroinflammation has been reported in the etiology of primary and secondary neurodegenerative diseases, such as Alzheimer’s disease (AD), Parkinson’s disease (PD), multiple sclerosis (SM), amyotrophic lateral sclerosis, Huntington’s disease (HD), stroke, and epilepsy [53, 54].

The main cellular effectors of neuroinflammation are astrocytes and microglia, as well as T lymphocytes, perivascular monocytes, and macrophages invading to the sites of insult from circulation. Microglial cells and astrocytes undergo activation in the process of microgliosis and astrogliosis, respectively [55]. The processes of microglia and astrocyte activation, T lymphocyte infiltration, and overproduction of a variety of inflammatory cytokines have been demonstrated in association with neuronal loss even during the pre-symptomatic phase of ALS [56].

Microglia are the first line of immune defense in the CNS. The cells have been shown to be highly plastic and could acquire distinctive phenotypes in response to various stimuli. Under physiological conditions, microglia retain a relatively quiescent phenotype. However, these cells constantly monitor local microenvironment and communicate with astrocytes and neurons by secreting anti-inflammatory molecules and neurotrophic factors like insulin-like growth factor 1 (IGF-1), transforming growth factor-β (TGF-β), brain-derived neurotrophic factor (BDNF), and nerve growth factor (NGF) [57]. Following injury or pathogen invasion and exposure to pro-inflammatory cytokines such as interferon-γ (IFN-γ), IL4 and tumor necrosis factor-α (TNF-α), microglial cells become polarized (activated) toward a pro-inflammatory phenotype. Upon activation, microglia promptly release an entirely different set of molecules, such as pro-inflammatory cytokines and chemokines, reactive oxygen species (ROS) or nitric oxide (NO), which contribute to the onset of local inflammatory response [58, 59].

The danger signals from damaged tissues include ATP released by dying and abnormally functioning neurons. The nucleotide-metabolizing enzymes are represented on microglial cells mainly by ecto-nucleoside triphosphate diphosphohydrolase 1 (ecto-NTPDase1, CD39) and ecto-5′-nucleotidase (CD73) [3]. Concerted action of these enzymes restores the balance between ATP and adenosine concentration that will be discussed in detail in the next chapters.

Astrocytes are the most abundant fraction of cells with a glial phenotype in the brain. Despite this population of cells has long been neglected or misidentified, astrocytes represent a key component in the brain environment. They provide neurotrophic factors, control synaptic functions and formation, regulate the concentration of neurotransmitters at synapses, and are involved in a wide range of homeostatic functions [60]. In the course of neurodegenerative diseases, including ALS, astrocytes become reactive by altering their morphology and molecular expression patterns [55, 61]. Activation of P2X7 receptor on spinal cord astrocytes was proved to trigger cytotoxic cascade and exert neurotoxic effect on motor neurons [62]. Accompanying processes involve induction of nuclear factor κ light-chain enhancer of activated B cells (NF-κB) and activator protein 1 (AP-1), and stimulation of cyclooxygenase-2 (COX-2) activity [3]. Another important astrocyte dysfunction in ALS is attributed to lowered release of neurotrophic factors, such as BDNF, glia-derived neurotrophic factor (GDNF), or vascular endothelial growth factor (VEGF). Neurotrophic factors are known to improve the neuron survival and repair; therefore, their deficiency significantly attenuates neuroregeneration [63].

Lymphocyte infiltration was described in ALS patients in the corticospinal tracts and anterior horns of the spinal cord [64]. T-helper CD4^+^ cells permeate at the onset of the disease and then accumulate during disease progression, whereas T-cytotoxic CD8^+^ cells are only present at the end stage of disease. In the ALS mice model, CD4^+^ T lymphocytes slowed disease progression, extended disease duration by 50%, modified the microglial phenotypes, and prolonged survival. This probably occurs by enhancing the expression of neurotrophic factors, such as IGF-1 and glial glutamate transporter-1 **(**GLT-1) [65]. At pre-symptomatic stage, treatment of ALS mice with CD4^+^ T lymphocytes significantly delays the onset of symptoms (action ascribed to CD4^+^ CD25^+^regulatory T-cells) and increases the latency between disease onset and entry into late stage (action mediated by CD4^+^ CD25^−^effector cells) [66, 67]. In the recent study, the expansion of endogenous regulatory T-cells in a mouse model of ALS significantly prolonged motor neuron survival time, suppressed glial cell immunoreactivity, and enhanced neuroprotective gene expression. Regulatory T-cells were also shown to correlate with a slower rate of disease progression in ALS patients [68].

Mast cells function as environmental “sensors” to communicate with other players under physiological conditions or during immune responses, based on their widespread tissue presence near blood vessels and surfaces exposed to the environment [69]. In the nervous system, mast cells represent an important peripheral counterpart in intercellular cross-communication. Upon activation, mast cells secrete numerous vasoactive, neurosensitizing, and pro-inflammatory mediators, including histamine, serotonin, cytokines, proteolytic enzymes (e.g., chymase, tryptase, acid hydrolases), lipid metabolites (prostaglandin D2, leukotriene C4, platelet-activating factor), neuropeptides, growth factors (NGF and VEGF), and nitric oxide [70]. Mast cells are also an abundant source of ATP which is stored in their granules and secreted upon activation [69]. Serum and CSF samples of ALS patients display elevated amounts of IL-12 and IL-15 [71], the latter cytokine acting as a mast cell chemoattractant, resulting in mast cells accumulation around degenerating motor neurons. Mast cell involvement in the neuromuscular junction denervation was recently investigated in an animal ALS model, where the authors observed a marked infiltration and degranulation of mast cells that correlated with disease progression [72].

The cross-talk between different cell types in the nervous system has a recognized role in neural information processing. Glia-neuron, mast cell-glia, and glia-glia cells communicate bi-directionally through the release of extracellular signaling molecules. Studies from the last decades have provided strong evidence for the complexity of this cross-talk [50, 69, 73]. The outcome of this intercellular communication is dependent on the local pathophysiological status, e.g., stress or injury, and on environmental factors, e.g., cytokine concentration. Molecules contributing to the intercommunication also include extracellular nucleotides, particularly ATP. All the cellular players can both release and simultaneously respond to the nucleotide signal via numerous purinergic receptors. Therefore, eATP generates a feedback loop that drives the persistent pro-inflammatory and detrimental response [3, 74, 75]. During the progression of ALS, microglia, astrocytes, and motor neurons enter in this pro-inflammatory cross-talk, followed by exacerbation of pathological processes. More insight into the communication between different cell types should provide important novel therapeutic approaches to promote repair and reduce neuroinflammation.

## The involvement of purinergic signaling in the ALS pathology

Purinergic signaling regulates glial proliferation, motility, survival, and myelination, as well as facilitates interactions between neurons and vascular and immune system cells [3, 73]. Numerous studies indicate that a vast interplay occurs also at the cell membrane among purinergic receptors, ecto-nucleotidases, and transporters, resulting in the insurgence or maintenance of neuroinflammatory conditions [73, 76]. In the following subsections, we will present in detail the contribution of individual purinergic signaling components in the pathogenesis of ALS and potential therapeutic directions (Table 2).

**Table 2:** Fundamental discoveries of purinergic contribution to ALS pathogenesis

Purinergic component		Contribution to ALS	References
Adenosine and A2A receptors	Ado	Significantly increased in the cerebrospinal fluid of ALS patients	[77]
	A2A	Receptors activation makes motor neurons susceptible to excitotoxic challenge	[78]
	A2A	Upregulated in lymphocytes from ALS patients	[79, 80]
	A2A	Upregulated in motor neurons from spinal cords of SOD1G93A mice and ALS patients	[80]
ATP and P2 receptors	P2X7	Microglial expression increased in post-mortem spinal cord samples from ALS patients	[81]
	P2X4, P2X7, P2Y6	Upregulated in SOD1-G93A mice microglia	[75]
	P2X7	Activation of microglial receptor induces cell death of ALS motor neurons	[75]
	P2X7	Activation of astrocytes receptor initiates motor neurons death in vitro	[62]
	ATP, BzATP	Small doses induce motor neuron death in vitro	[82]
	P2X7	Activation of receptor by BzATP up-regulates the miRNAs transcriptome in SOD1-G93A microglia	[15]
	P2X7	Ablation of receptor in SOD1-G93A mice aggravates gliosis and motor neuron death	[83]
	P2X7	Receptor antagonist Brilliant Blue G ameliorates spinal cord pathology in SOD1-G93A mice	[84]
	P2X4	Upregulated in degenerating motor neurons in ALS mouse and rat spinal cord	[12, 85]
	P2Y12	Expression progressively reduced in SOD1-G93A mice microglia	[86, 87]
	P2X4, P2X7	Upregulated in sciatic nerves of SOD1-G93A mice	[9]
Enzymes	CD39	Gene expression downregulated in spinal cord microglia from SOD1G93A mice and ALS patients	[88]
	CD39	Protein expression downregulated in SOD1-G93A mice microglia	[9]
	ADK	Increased activity in reactive astrocytes decreases adenosine concentration and triggers neurodegeneration	[89]

### ATP and P2 receptors in ALS

#### P2X7

Injury to motor neurons caused by pro-inflammatory factors released by activated microglia is considered as one of the most critical pathogenic mechanisms in ALS. Among purinergic receptors, the P2X7 receptor was shown to be involved in chronic pain, neurodegeneration, and neuroinflammation [90]. This purinergic receptor is predominantly expressed on microglia and oligodendroglia, and at lower level on astrocytes [37]. P2X7 receptor activation mediates the release of IL-1 family cytokines including IL-1α, IL-1β, and IL-18 [91]. Release of IL-1β results in upregulation of proteins contributing to inflammatory processes, including COX-2, nitric oxide synthase (NOS), TNF-α, pro-caspase 1 as well as matrix metalloproteinase-9 (MMP-9), and cannabinoid receptor 2 (CB2). Interestingly, a significantly increased immunoreactivity of P2X7, together with COX-2 and CB2, has been shown in active microglia from post-mortem spinal cord samples of sALS patients and in the mSOD1G93A transgenic rodent model of fALS [81, 85, 92]. The P2X7 receptor-mediated neurotoxicity was also confirmed in mSOD1 astrocytes [62]. P2X7 receptor alone can activate cell death via both apoptotic and necrotic mechanisms leading ultimately to the development of neurotoxic phenotype. For instance, a peroxynitrite-fueled apoptotic cascade activated by P2X7 results in motor neuron death due to trophic factor deprivation, activation of p57 neutrophin death receptor (p75NTR), and expression of mutant forms of SOD1 [62, 82].

Emerging evidence indicates that P2X7 may have a dual function in onset and progression of ALS, either trophic and anti-inflammatory or toxic and pro-inflammatory. The fact that ablation of P2X7 in SOD1-G93A mice exacerbates gliosis and motor neuron death at end stage of the disease supports a neuroprotective role of this receptor [83]. On the other hand, the activation of microglial P2X4, P2X6, and P2X7 receptors by 2′-3′-O-(benzoyl-benzoyl) ATP (BzATP), a non-hydrolysable form of ATP, leads to increased content of pro-inflammatory molecules such as TNF-α and COX-2 with the consequent motor neuron injury [75]. Moreover, the activation of P2X7 receptor by BzATP enhanced ROS production in SOD1-G93A mouse microglia through the activation of NADPH oxidase 2 (NOX2) which is the main ROS-producing enzyme in microglial cells and well-recognized player in the pathogenesis of ALS. Importantly, this microglia-mediated mechanism for motor neuron damage was shown to be prevented by ablation of P2X7 and the use of P2X7 specific antagonists such as A839977, A438079, and Brilliant Blue G (BBG) [93]. Other pathological mechanism by which P2X7 can exacerbate a neurotoxic phenotype of ALS microglia is miRNA dysregulation. Parisi and colleagues have shown that stimulation of P2X7 receptor by BzATP hyperactivated inflammatory miRNAs, such as miR-155, miR-125b, and miR-146b which are known to be upregulated in SOD1-G93A microglia. It resulted in downregulation of IL-6/STAT3 signaling and enhancement of TNF-α production, switching eventually microglia toward a detrimental phenotype [15].

It is assumed that the use of neuroprotective agents such as COX-2 inhibitors, P2X7 receptor antagonists, and CB2 agonists might exert beneficial effects in ALS pathology by slowing down the progressive damage to motor neurons [81, 92]. The P2X7 antagonist BBG is considered as a promising candidate for blocking the detrimental effects of P2X7 activation due to its high selectivity, low toxicity, and ability to efficiently cross the blood–brain barrier. Other P2 antagonists such as oxATP (oxidized ATP) and PPADS (pyridoxal-phosphate-6-azophenyl-2′,4′-disulfonic acid) fail to meet all of the above requirements [94, 95]. Brilliant Blue G has previously been proven to reduce neuroinflammation in traumatic brain and spinal cord injury [95–97], cerebral ischemia reperfusion [98], neuropathic pain [99], and in experimental autoimmune encephalitis [100]. The neuroprotective effects of BBG have been tested in the SOD1-G93A ALS mouse model at different phases of ALS to elucidate the role of P2X7 receptor in microglial polarization and neuroinflammation. Administration of BBG, beginning at a late pre-symptomatic phase, improved motor neuron performance and slightly delayed ALS onset, although with no effect on life span. Importantly, BBG neuroprotection occurred within a specific time frame, since BBG administration at earlier phases did not prevent disease progression [84]. In particular, BBG treatment enhanced motor neuron survival and significantly reduced microgliosis by downregulating the expression of pro-inflammatory proteins, NF-κB, and microglia markers of activated phenotype (NOX2 and IL-1β), and simultaneously upregulating other markers (IL-10 and BDNF). The key conclusion drawn from these results might be “the double face” of P2X7 receptor and the existence of a narrow therapeutic window concerning its beneficial role in ALS. The dual action of P2X7 during ALS progression seems to correspond to the switch of microglia from protective to lethal phenotype.

Most recently, the role of P2X7 receptor in microglia polarization has been confirmed in LPS-induced cellular model of inflammation. It was shown that P2X7 inhibition by the selective antagonist A438079 suppressed microglia activation [101]. Based on these findings, P2X7 has emerged as a potential marker of activated microglia. It is postulated that the microglial polarization switch is closely linked with the time when P2X7 starts to play a critical role in regulating neuroinflammation in ALS [9]. This hypothesis is strongly supported by the fact that ALS pathogenesis consists of two distinct neuroinflammatory stages. The first stage is neuroprotective due to the concerted action of regulatory T-cells, anti-inflammatory macrophages/microglia, and T helper cells, which sustain motor neuron viability by providing anti-inflammatory agents. As the disease and motor neuron injury accelerate, the second rapidly progressing stage emerges when activation of pro-inflammatory T-cells and macrophages/microglia leads eventually to neurotoxicity [9, 59, 102].

#### P2X4

A strong P2X4 immunoreactivity was shown to be associated with degenerating motoneurons in spinal cord ventral horn samples from mSOD1G93A rats. In parallel, P2X4 immunostaining detected degeneration in other neuronal populations, including noradrenergic neurons in the locus coeruleus, Purkinje cells in the cerebellum and serotonin containing neurons in the raphe nucleus [85]. More interestingly, the P2X4 antibodies were able to recognize neurotoxic species of misfolded SOD1G93A in motor neurons but not in glial cells. It was suggested that neuronal P2X4-immunoreactive SOD1G93A conformers may have a pathogenic role in the promotion of neuroinflammation since they activated microglia and astroglia when injected intracerebrally into normal animals [103].

Upregulation of P2X4, both at mRNA and protein level, was also found in ALS microglia from SOD-G93A mice [75]. Most recently, Volonté and colleagues have demonstrated for the first time the increased expression of P2X4 and P2X7 proteins in the peripheral nervous system of SOD1-G93A mice, namely in sciatic nerves, and nominated these P2 receptors as potential diagnostic biomarkers for ALS at the peripheral level [9]. Finally, P2X4 receptor activation was shown to protect motor neurons. α-Amino-3-hydroxy-5-methyl-4-isoxazole propionic acid (AMPA) receptor-mediated excitotoxicity is considered as an important mechanism for motor neuron death in ALS. Andries and colleagues showed that preincubation of motor neurons with P2X4 allosteric modulators such as ivermectin (anti-parasite medication) and Cibacron Blue 3G-A (CB) protected the cells against kainate-induced excitotoxicity in vitro. In addition, ivermectin potentiated the protective effect of low ATP concentrations to motor neurons, presumably by increasing the number of P2X4 molecules on the cell surface, and most importantly, extended survival of SOD1-G93A mice by almost 10% [12].

#### P2Y6

P2Y6 receptor is activated mainly by UDP, partially sensitive to UTP and ADP, and completely insensitive to ATP. P2Y6 was shown to be upregulated in SOD1G93A mice microglia; however, the role of this receptor in ALS remains elusive [75]. Likewise P2Y2 and P2Y4 receptors, P2Y6 regulates microglial phagocytosis upon activation by UDP [104]. It was suggested that this process may play important role in facilitating the uptake of cellular debris generated after neuronal damage. In fact, damage of hippocampal neurons by kainate in vivo and in vitro resulted in upregulation of microglial P2Y6 expression and consequent activation of microglial phagocytic activity via UDP/P2Y6 signaling [105].

#### P2Y12

The involvement of P2Y12 in microglia process dynamics is well established, including its key role in the regulation of microglia activation, chemotaxis, and migration [86, 106]. This purinergic receptor can serve as a marker of a resting/surveillant branched state of ALS microglia as well as a marker distinguishing CNS-resident microglia from blood-derived macrophages infiltrating CNS upon neuronal injury [87, 107]. Moreover, a dramatic and continuous reduction of P2Y12 receptor expression was observed after microglia activation following brain injury [106]. Consistently, P2Y12 expression was found to be progressively reduced in spinal cord microglia of SOD1-G93A mice and ALS patients during neuroinflammation [87, 107]. Interestingly, P2Y12 expression was significantly decreased at symptomatic stage, when the disease accelerates, and completely lost at end stage of the disease when motor neuron loss, oligodendrocyte degeneration, and microglia activation are known to be augmented [87]. These findings indicate a neuroprotective action of P2Y12 at early stage of ALS that is consistent with results obtained by Apolloni and colleagues. The authors showed that short treatment (from asymptomatic to symptomatic phase) with antihistamine drug clemastine reduced disease progression and improved survival of SOD1G93A ALS mice by enhancing anti-inflammatory phenotype of microglia via upregulation of P2Y12 together with P2Y7, arginase-1 and CD1639. Because long treatment with clemastine (from asymptomatic until the end stage) failed to ameliorate ALS progression, the beneficial effects of this drug are thought to be tightly dependent on the first phase of neuroinflammation in ALS, characterized by neuroprotective functions of microglia [84].

### Adenosine and P1 receptors in ALS

In 1999, Yoshida and colleagues reported a significantly increased adenosine concentration in the cerebrospinal fluid of progressing ALS patients; however, neither diagnostic nor prognostic potential of these findings had been evaluated [77]. Adenosine, formed as the product of eATP degradation, possesses neuroregenerative potential. In ALS patients, overexpression of adenosine kinase (ADK) is a common pathologic hallmark, and the consequent increase in astrocyte ADK activity disrupts adenosine homeostasis triggering ultimately neurodegeneration [89]. On the other hand, adenosine is capable to stimulate astrogliosis by activating P1 receptors on astrocytes. A2A and A3 receptors are involved in the astrogliosis initiation, whereas the activation of A1 receptor inhibits the proliferation of astrocytes [3, 108]. Moreover, in vitro studies revealed that adenosine at physiological concentration of about 10 nM activates A2A receptor in astrocytes, followed by affecting GLT-1 and inhibiting glutamate uptake [3]. And ALS patients and SOD1-G93A mice suffer from suppressed glutamate uptake that drives excitotoxic processes and motoneuron degeneration [109].

The first evidence for the involvement of A2A in ALS was provided by A2A blockage with the selective antagonist KW6002, which protected motor neurons from toxic insult triggered by the expression of mutant versions of SOD1 and dynactin subunit p150^glued^. The neuroprotective effect of A2A antagonism was attributed to attenuated tyrosine receptor kinase B (TrkB) signaling, known to induce vulnerability of motor neurons to excitotoxic challenge upon activation by BDNF. Moreover, a physical interaction between A2A and TrkB within lipid rafts of motoneurons was shown to be prerequisite for TrkB transactivation [110]. On the contrary, activation of A2A in the absence of BDNF signaling rendered these cells susceptible to excitotoxicity [78]. However, the above findings are not consistent with results gained from studies on motor neuron cultures exposed to BzATP and in SOD1-G93A mouse model for ALS. Namely, it was shown that high doses of adenosine protect motor neurons from death induced by BzATP, whereas low doses exert no beneficial effect. Because adenosine production results from rapid ATP breakdown, it was concluded that ATP at high concentrations (1 mM), paradoxically, may be protective to motor neurons, while at low concentrations trigger neuronal apoptosis upon P2X7 activation [82]. A protective role of A2A has been also demonstrated in SOD1G93A mice model, as treatment with the selective agonist CGS21680 resulted in delayed disease onset [111], whereas intake of caffeine, a nonselective P1 receptor antagonist, significantly decreased survival time of ALS animals [112].

A possible involvement of the P1 receptors in ALS has also been investigated at peripheral level. Expression profiling of all P1 receptor subtypes revealed a significant upregulation of A2A in lymphocytes from ALS patients, compared to healthy subjects, and a positive correlation between A2A density values and scores of the revised ALS Functional Rating Scale, which is a useful predictor for ALS progression. Moreover, activation of A2A by the selective CGS21680 agonist led to increased production of cyclic AMP in lymphocytes from ALS patients as compared with a control group. The negative correlation between A2A density and the severity of disease symptoms, which highlights a possible role for these receptors in immunosuppressive responses in ALS, might also represent a promising perspective for alternative therapeutic approaches for ALS based on modulation of A2A receptor activity [79]. In view of anti-inflammatory effects of A2A activation to peripheral immune cells, these findings support a neuroprotective role of A2A in ALS, at least at peripheral level.

The most recent discovery has provided evidence for a more complex role of A2A-mediated adenosine signaling in ALS. Firstly, it was shown that A2A was upregulated in motor neurons from spinal cords of symptomatic SOD1G93A mice and end-stage ALS patients [80] that was in agreement with previous studies reporting the increased expression of A2A in motor neurons of end-stage SOD1G93A mice [112]. Furthermore, a direct treatment of adenosine at concentration of 0.3 or 1.0 μM induced death of embryonic stem cell-derived motor neurons (ESMN) cultured in vitro suggesting that A2A blockage might be neuroprotective to motoneurons. The subsequent A2A inhibition by the selective KW6002 antagonist and partial genetic ablation of A2A efficiently protected ESMN from SOD1G93A+ astrocyte-induced cell death and slowed disease progression of SOD1G93A mice [80]. In light of these studies, a novel toxic effect of adenosine on spinal cord motor neurons has been discovered; however, the exact mechanism of this adenosine-induced A2A activation is elusive and merits further investigation.

Contrary to A2A, the role of other P1 receptors in ALS still remains elusive, but evidence for a loss of A1-A2A functional cross-talk at the neuromuscular junction in pre-symptomatic SOD1G93A mice has been reported recently. A1-mediated adenosine signaling may contribute to exacerbation of the disease during the symptomatic phase when A1 tonic activation was shown to be enhanced [113].

### NTPDases

CD39 (NTPDase1) is exclusively expressed on the surface of microglia where it plays a predominant role in the inactivation of P2 receptor-mediated signaling by catalyzing a rapid ATP degradation to ADP and AMP [114]. Consequently, any changes in CD39 expression may result in disturbed nucleotide homeostasis and subsequent alterations in purinergic transmission. In fact, CD39 downregulation at mRNA level has been demonstrated in microglia from the spinal cord of SOD1-G93A mice and ALS patients [88], which was further confirmed at protein level in cortical primary microglia from SOD1-G93A newborn mice [9]. CD39 downregulation caused prolonged activation of P2 receptors as a consequence of a significantly reduced hydrolysis of extracellular ATP. As three ATP-sensitive P2 receptors, namely P2X4, P2X7, and P2Y6, are found to be upregulated in ALS, the above findings emphasize a critical role of CD39 in the regulation of neuroinflammatory events mediated by ALS microglia [75]. In general, dysregulation of any purinergic element along with altered concentrations of signaling agents in the extracellular milieu leads to the enhancement of inflammatory responses.

CD39L1 (NTPDase2) was found to be highly expressed in hippocampal, cortical, and cerebellar astrocytes where it plays a predominant role in regulation of the ATP/adenosine balance. Consequently, impairment of its activity can increase eATP concentration resulting in activation of P2X receptor and initiation of inflammatory astrogliosis, leading ultimately to neuronal cell death. For this reason, NTPDase2 is considered as another potent therapeutic target in human CNS disorders, but its precise role in ALS needs to be elucidated [115].

**Fig. 3:**
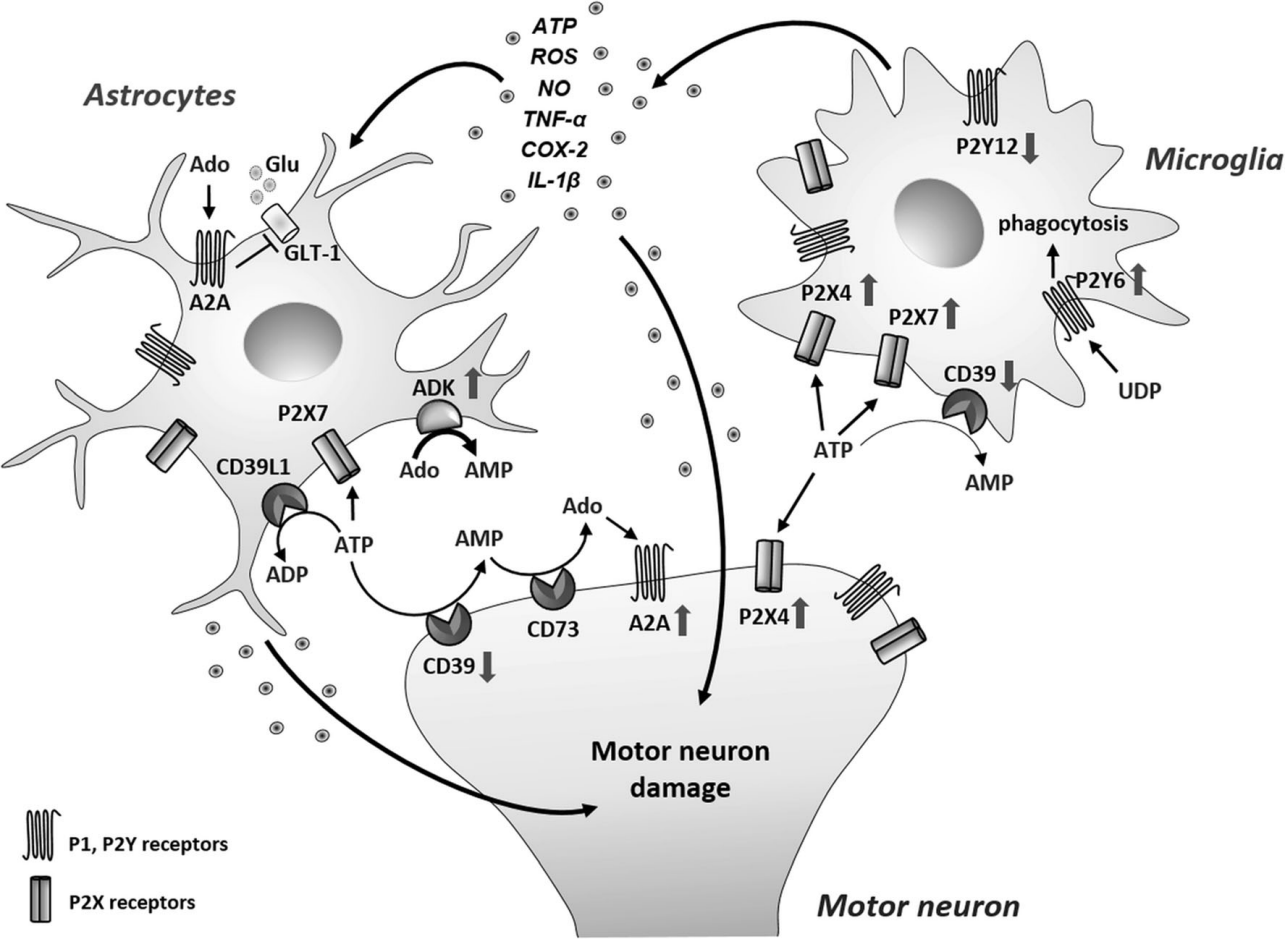
Purinergic dysregulation in ALS

To summarize the current findings discussed in the above chapter, in Fig. 3, we depict a network of purinergic components and mechanisms recognized so far to contribute to aberrant neuron-glia communication and enhanced neuroinflammation underpinning ALS pathogenesis.

## Current and potential treatment for ALS

Despite intensive research, little in the field of new effective therapeutics for ALS has been developed so far. The most frequently provided reason for explaining the difficulties in finding a remedy for curing or slowing the progression of ALS is that the disease is multi-genic, multi-factorial, and multi-systemic. Till 2016, more than 50 major phase II or III clinical trials in ALS patients have been reported to fail, despite positive results from animal models [116]. In this context, the question arises whether the most widely used SOD1 transgenic mice models properly reflect different aspects of ALS heterogeneity in patients with both sALS and non-SOD1 linked fALS. It is believed that the latest discoveries in genetics of ALS, including mutations in *C9ORF72* and *TBK1* genes, will open new perspectives in the generation of relevant animal models for ALS, with consequent potent contribution to the development of new biomarkers and therapeutic targets for the disease [117].

For the past 22 years, the only available FDA-approved intervention for ALS has been riluzole (brand names Rilutek or Teglutik). However, this anti-excitotoxic drug extends patient’s life span by only several months without improving muscle strength and neurological function and is ineffective in later stages of the disease [118]. On May 5, 2017, the second drug, known as either edaravone (Radicava) or MCI-186, was finally approved by the FDA for ALS treatment [119]. Edaravone administrated intravenously once a day for 14 days, followed by a 2-week break, decreases disease progression in early-stage ALS patients with no respiratory involvement and indications for gastrostomy [119]. This anti-oxidant and free-radical scavenger delays motor neuron degeneration but provides limited survival benefit [120, 121]. Among other compounds currently being tested in clinical trials, arimoclomol is considered as a promising therapeutic candidate. This so-called smart drug induces the expression of heat shock proteins (HSP) exclusively under cellular stress conditions. It results in increased capacity of protein quality control and degradation of misfolded proteins, including mutant SOD1. Administered to SOD1-G93A mice after the onset of symptoms, arimoclomol slowed disease progression, increased survival, and improved muscle function. The drug is currently tested in phase III clinical trial [121, 122].

## Purinergic signaling as potential therapeutic target in ALS

A growing body of research provides evidence for an essential contribution of purinergic signaling to the development of many neuroinflammatory and neurodegenerative diseases. Most importantly, some results of these studies have been already implemented in the treatment of Parkinson’s disease (A2A receptor antagonist istradefylline) [123], cerebral ischemic stroke (P2Y12 receptor antagonists clopidogrel and ticlopidine, and adenosine transporter inhibitor dipyridamole) [124, 125]. It is also believed that they might contribute to establishing new treatment strategies for multiple sclerosis, epilepsy, migraine, and neuropathic pain [53, 126–128]. Since the discoveries of microglia activation as an important mechanism of motor neuron death in ALS and extracellular ATP as a crucial neuron-to-glia alarm signal, the involvement of purinergic signaling in neuroinflammation has become evident and largely accepted.

During the progression of ALS, microglia, astrocytes, and motor neurons continue the pro-inflammatory cross-talk, inter alia through P2X7 activation that was shown to be prevented by P2X7 antagonists [62, 75]. The P2X4 receptors, however, exert protective effects in motor neurons. Considering these results, it can be concluded that low ATP concentrations protect cells against excitotoxic stimuli through P2X4 receptors, whereas high concentrations of ATP produce toxic P2X7 activation. Finally, adenosine is also involved in ALS progression since adenosine A2A receptor antagonists prevent motor neuron death at the symptomatic phase [73, 110]. In light of current findings, P2X7 and A2A are recognized as dual-function purinergic receptors, which course of action closely depends on ALS state, particularly neuroinflammatory landscape of the disease. However, despite many lines of evidence for essential contribution of purinergic signaling to motor neuron damage, it is important to emphasize here that there are currently no clinical trials targeting the purinergic players in the therapy of ALS. In terms of the high complexity of the purinome comprising diverse nucleotide- and nucleoside-metabolizing enzymes and purinergic receptors differentially distributed on CNS cell types, further intense research has to be performed to elucidate various implications of the purinergic signaling in ALS and evaluate its therapeutic potential in the fight of this relentlessly progressive disease. However, based on the knowledge gained so far, the therapeutic significance of the following strategies might be further evaluated with reference to ALS: (a) quenching the pro-inflammatory function of P2X7, (b) enhancing the anti-inflammatory action of A2A, (c) modulating the cell surface expression of purinergic receptors, and (d) regulating the activities of nucleotide-metabolizing ecto-enzymes, in particular CD39.

## Conclusions and future perspectives

Despite many research efforts to decipher ALS pathogenesis, recent years have not produced a breakthrough both in explaining the ALS etiology and developing new effective therapeutic therapies. Composite etiology along with the lack of specific biomarkers as well as an insufficient knowledge of risk factors for ALS hinder accurate diagnostics, especially in the early stages of this invariably fatal disorder. Hence, further studies should focus on understanding the primary mechanisms triggering motor neuron degeneration, elucidating mechanisms by which SOD1 mutations cause neuronal death, identifying novel genes and pathways associated with ALS, establishing new animal models for the disease, searching for early and selective diagnostic biomarkers, and discovering new, effective strategies to prevent the insurgence and progression of ALS [3, 129].
